# Editorial: Social psychological perspectives on threat: understanding climate, economic, and health threats

**DOI:** 10.3389/fpsyg.2026.1936785

**Published:** 2026-07-31

**Authors:** Ginés Navarro-Carrillo, Juan Carlos Oyanedel, Joonha Park

**Affiliations:** 1 Mind, Brain and Behavior Research Center (CIMCYC), Department of Social Psychology, University of Granada, Granada, Spain; 2 Faculty of Education and Humanities, Universidad Andres Bello, Santiago, Chile; 3 Graduate School of Education, Kyoto University, Kyoto, Japan

**Keywords:** climate change, climate threat, COVID-19, economic threat, health threat

## 1 Introduction

Crises and periods of marked instability, such as the climate emergency or the recent COVID-19 pandemic, impact more than just non-psychological indicators. Their effects, driven by the psychological threat they impose, extend across multiple psychosocial domains, as recent research suggests (e.g., Pizarro et al., 2024; Shanaah et al., 2025). Furthermore, counter to intuitive expectations, these psychosocial outcomes are not always adverse. Evidence indicates, for instance, that such effects can trigger adaptive, other-oriented responses (Alonso-Ferres et al., 2020; Fridman et al., 2022).

Undoubtedly, the scale of recent global crises has significantly stimulated empirical interest in various forms of psychological threat, including those at the core of this Research Topic (i.e., climate, financial, and health-related threats). However, further research is needed to better understand the diverse potential effects and underlying psychological processes associated with these threats.

This Research Topic comprises 19 articles that utilize diverse methodologies to better characterize these experiences of psychological threat, their correlates, and, in several instances, the psychological mechanisms underlying their effects. To provide a comprehensive overview of these studies while embedding them theoretically within a broader framework, we categorize the contributions into the following thematic axes based on their primary object of analysis: (a) climate-related perceived threats; (b) financial-related perceived threats; (c) health-related perceived threats; and (d) other domains of perceived threat.

Specifically, of the 19 articles in this compendium, the majority analyze phenomena linked to financial threat (seven manuscripts) and health threat (seven manuscripts), while only one examines psychological factors associated with climate threat. Finally, two articles investigate processes simultaneously related to multiple threat experiences, whereas the remaining two examine aspects tied to other types of psychological threat.

## 2 Climate-related perceived threats

In the only paper addressing climate threat, Diaz-Silveira et al. conduct a series of four studies focusing on how individuals cope—both emotionally and behaviorally—with the climate crisis. This area has received less academic attention than the assessment of its psychological repercussions.

The authors developed and validated the Climate Emergency Coping Scale (CECS), a psychometrically robust measure comprising three factors: (a) functional-individual coping, which includes individual actions aimed at managing one's emotions and contributing to climate action; (b) functional-social coping, which encompasses social, collective, and interpersonal coping strategies; and (c) dysfunctional coping, defined by actions that benefit neither the individual nor the environment, typically involving maladaptive responses (e.g., avoidance, resignation, or denial). Interestingly, Diaz-Silveira et al.'s work demonstrated that higher levels of eco-anxiety and eco-worry indicated a greater tendency to endorse functional-individual and functional-social coping actions. Furthermore, the authors found that eco-anxiety and eco-worry exhibited a divergent pattern of correlations with dysfunctional coping strategies: while eco-anxiety was positively associated with these strategies, eco-worry was negatively related to them. However, it is worth noting that these latter correlations were weak. Taken together, Diaz-Silveira et al.'s findings suggest that eco-anxiety and eco-worry may act as catalysts for positive coping strategies at both the individual and collective levels.

## 3 Financial-related perceived threats

The papers in this section examine how various aspects of the psychological experience of financial threat affect multiple psychosocial outcomes, ranging from prosocial behavior (Stollberg et al.), individual economic decisions (Hua and Mi; Hung et al.), and workplace experiences (Ding and Wang) to entrepreneurial intentions (Gebresilase et al.) and wellbeing (Canal-Serantes et al.; Wu et al.).

Regarding prosocial behavior, Stollberg et al. conducted an experimental investigation to determine whether the salience of economic and humanitarian crises could mitigate or reverse the negative effects of neoliberal beliefs on other-oriented behavior. In Study 1, participants were exposed via a fictitious newspaper article to an economic threat that violated shared values of equality and fairness (vs. a non-salient economic threat condition). This manipulation effectively buffered the negative association between neoliberal beliefs and various general prosociality measures. In a second study, designed as a replication within a humanitarian crisis context (the war in Ukraine), the authors presented a video detailing the humanitarian consequences of the Russian invasion of Ukraine (vs. a neutral, non-threatening video condition), while also assessing participants' willingness to help Ukrainians—an out-group prosocial measure. Their results were consistent with the preceding study: the humanitarian threat salience condition led to an increased tendency to help Ukrainians. Although this main effect was independent of neoliberal beliefs, the results showed no interaction between the experimental manipulation and neoliberal beliefs in predicting solidarity toward Ukrainians. Thus, Stollberg et al.'s findings suggest that economic threat salience specifically may buffer the negative effects of neoliberalism on prosocial behavior.

The studies by Hua and Mi and Hung et al. focus on individual economic decisions through different theoretical lenses. Hua and Mi argue that the long-term economic and emotional consequences of the COVID-19 pandemic influence daily life. Specifically, they empirically examined the role of perceived declining income in household saving and consumption behavior. Their results showed that participants experiencing income decline exhibited a greater propensity for low-risk saving behaviors and anticipatory consumption. Conversely, residents with stable or increasing incomes tended to maintain riskier saving and investment behaviors and plan for long-term consumption. Interestingly, scarcity anxiety mediated the relationship between perceived declining income and these saving/consumption decisions. Hung et al. used an experimental approach to study the compromise effect—the tendency to select the middle option among three alternatives because it is perceived as the safest. They investigated how credit card use (vs. cash payment) influences this effect during purchasing decisions. The authors demonstrated that credit card payments reduce the compromise effect compared to cash payments. Furthermore, the pain of paying mediated the relationship between the payment method and the compromise effect.

Social comparison plays a prominent role in the work of Ding and Wang and Wu et al.. Ding and Wang explored the role of upward social mobility in Chinese women's workplace experiences. After analyzing 4,043 comments on the Zhihu platform, the authors suggest that women's upward social mobility in China may be constrained by both structural factors (salary, discrimination, and geography) and personal factors (education, individual agency). Wu et al. focused on wellbeing, identifying wealth comparisons across different levels of social distance (family, friends, and the internet) as potential determinants. Their results confirmed that wealth comparisons decrease subjective wellbeing, with the most pronounced effect occurring for comparisons with friends, followed by family and the internet. Additionally, their findings suggested that stress helps explain these negative effects, whereas income—along with life satisfaction—may buffer this adverse impact.

Wellbeing is also the primary focus of Canal-Serantes et al. Across multiple studies, the authors analyzed the linkages between financial threat and various wellbeing and health indicators. As expected, their results showed that higher financial threat predicted poorer wellbeing and health outcomes. By incorporating health threat in Studies 2 and 3, they found that it was similarly associated with diminished wellbeing and health. Interestingly, Canal-Serantes et al. revealed that perceived control across different life domains (close relations, health, work, and finances) differentially mediated these effects: perceived personal control over health, work, and finances mediated the effects of financial threat on wellbeing, whereas perceived control over relations and health mediated the association between health threat and wellbeing.

Finally, Gebresilase et al. also analyzed control-related variables—specifically, internal and external locus of control—in Ethiopia, a context where financial threats are particularly salient. The authors examined the associations between locus of control and entrepreneurial intentions, which, as theorized, can serve as a precursor to economic development in such environments. Their results showed that an internal locus of control was positively associated with entrepreneurial intentions, whereas an external locus of control displayed the opposite pattern.

## 4 Health-related perceived threats

The majority of the articles within this category are situated in the context of the COVID-19 pandemic to analyze phenomena related to, among other aspects, the risk perception of severity or death from the pandemic (Rosa et al.), fear of COVID-19 (Minzenberg and Yoon), the progression of obsessive-compulsive symptoms (Costa et al.), individuals' perceptions of social security (Wang and Zhang), or digital governance and policy compliance (Huo et al.). The remaining articles examine other psychosocial factors of interest regarding various public health emergencies or problems (Teke and Karaman; Wang et al.).

Regarding the risk of severity or death from the pandemic, Rosa et al. conducted a systematic review—with a total of 19 articles included—to study associated factors. Their findings indicated that the perceived risk of COVID-19 severity or death may vary based on different indicators. More specifically, Rosa et al. found that the perceived risk of severity or death from COVID-19 was frequently associated with the following characteristics: (a) age (the older the age, the greater the risk); (b) gender (higher risk among females); (c) unfavorable personal experiences associated with COVID-19 (the more negative personal or family experiences, the greater the risk); (d) the presence of chronic non-communicable diseases (e.g., the greater the presence of high-risk medical conditions, the greater the perceived risk for COVID-19); and (e) educational attainment (the lower the educational level, the greater the risk).

Minzenberg and Yoon included a psychological variable closely connected to health threat and the perceived risk analyzed by Rosa et al.: the fear of COVID-19. Their results showed that higher levels of fear of COVID-19 were associated with greater experience of affective symptomatology (i.e., anxiety, depression, and paranoia), as well as higher levels of xenophobia and conspiracy mindedness, although the correlations with these latter two variables yielded smaller effect sizes. On one hand, these data reveal the detrimental psychological functioning associated with the fear of COVID-19. On the other hand, their results also demonstrated that fear of COVID-19 clustered alongside the aforementioned affective symptoms (i.e., anxiety, depression, and paranoia) into a single factor termed psychopathology, which, interestingly, was related to a greater inclination toward adaptive health behaviors.

However, some of the adaptive responses aimed at containing the spread of COVID-19 might, in fact, overlap with behaviors associated with disorders such as obsessive-compulsive disorder (Costa et al.). For this reason, these authors examined the longitudinal effects of COVID-19 on, among other aspects, obsessive-compulsive symptoms in a sample from the general Portuguese population. Regarding washing behaviors, their results showed an initial peak in 2020 (the first wave of assessment, coinciding with the first lockdown), followed by a progressive decrease in these levels across the two subsequent assessment points in 2021 and 2022. Consequently, the authors argue that this initial increase may represent an adaptive response to the threat posed by the pandemic, while the subsequent reduction aligns with the decrease in perceived threat and the gradual lifting of restrictive measures.

Also within the context of COVID-19, Wang and Zhang a used a representative Chinese sample to analyze the role of perceived social justice (at the levels of opportunity and outcome) and political trust in the sense of social security. Their findings confirmed that higher scores in these social justice variables (opportunity fairness and outcome fairness) indicated a greater sense of social security. Furthermore, their results revealed that political trust mediated the association between perceived social justice and social security.

Focusing primarily on the COVID-19 pandemic context in China and using a mixed-methods approach, Huo et al. examined the role of digital governance in public policy compliance. Their results revealed that higher levels of perceived usefulness, ease of use, and transparency regarding digital government systems were associated with a greater tendency to comply with public policies.

In a different public health emergency context—specifically, the psychological impacts of a natural disaster—Teke and Karaman examined a series of risk and protective factors for life satisfaction following the earthquake of February 6, 2023, in Turkey. More specifically, they analyzed the associations among perceived stress, hope, irrational beliefs, and life satisfaction. Their results showed that increased perceived stress following the earthquake was related to more irrational beliefs, less hope, and lower life satisfaction. Moreover, their data revealed that irrational beliefs and hope mediated the relationship between stress and life satisfaction.

Finally, Wang et al. focused on the study of non-suicidal self-injury, a public health issue affecting adolescent populations. In general, these authors examined the relationships among school bullying victimization (which constitutes a risk factor), rumination, friendship quality, and non-suicidal self-injury. Their results revealed that adolescents who experience bullying appear to show a greater inclination toward non-suicidal self-injury, a relationship mediated by an increased tendency to ruminate. Importantly, the relationship between rumination and non-suicidal self-injury was buffered by higher friendship quality.

## 5 Other domains of perceived threat

This category includes two studies examining composite threat or risk factors comprised of multiple simultaneous threat sources (Gong et al.; Kolesovs), and two others focusing on distinct forms of psychological threat (Huang et al.; Jiménez-Moya et al.).

Kolesovs analyzed the contribution of a composite psychological threat factor (comprising perceived epidemiological, ecological, and economic threats) to the sense of belonging and wellbeing within a sample of Latvian participants during the first wave of the COVID-19 pandemic. Although the results showed that this global threat factor had no direct effect on life satisfaction, a direct effect was observed via an increased sense of relational belonging. Consequently, as Kolesovs notes, this finding suggests that the strengthening of a sense of belonging in response to perceived psychological threats may represent a form of adaptive social response.

Gong et al. conducted a meta-analytic study including 50 papers to examine the relationship between risk perception (encompassing natural disasters, public health emergencies, accidents, and social security emergencies) and information-seeking behavior. Their results demonstrated that higher perceived risk was associated with a greater tendency to seek information relevant to these emergency situations. However, their data indicated that this relationship varied depending on the type of emergency, with positive and stronger effects observed for public health emergencies, followed by natural disasters.

Finally, Huang et al. and Jiménez-Moya et al. focused on personal identity and gender identity threat, respectively. Regarding the role of personal identity—conceptualized as the sense of self-continuity and coherence over time—Huang et al. found that higher personal identity was associated with lower levels of trait anxiety. On the other hand, the results of the experimental study by Jiménez-Moya et al. suggested that daily choices counter to gender stereotypes may trigger the activation of gender identity defense mechanisms that ultimately reinforce attitudes bolstering gender inequality.

## 6 Final considerations

In conclusion, the diverse approaches characterizing the 19 manuscripts in this compendium provide valuable insights into the role of psychological threats that are especially relevant in contemporary societies. We hope that this accumulated knowledge can be translated into more effective concrete actions or policies to address the challenges posed by crises and emergencies that generate psychological threat. Finally, we would also like to express our gratitude for the collaboration of the authors and reviewers involved in this Research Topic.
